# Supplementary figures and images for: Irrelevant auditory and tactile signals, but not visual signals, interact with the target onset and modulate saccade latencies
Source: PLoS One. 2020 Feb 11;15(2):e0221192. doi: 10.1371/journal.pone.0221192 (PMC7012454; doi:10.1371/journal.pone.0221192)

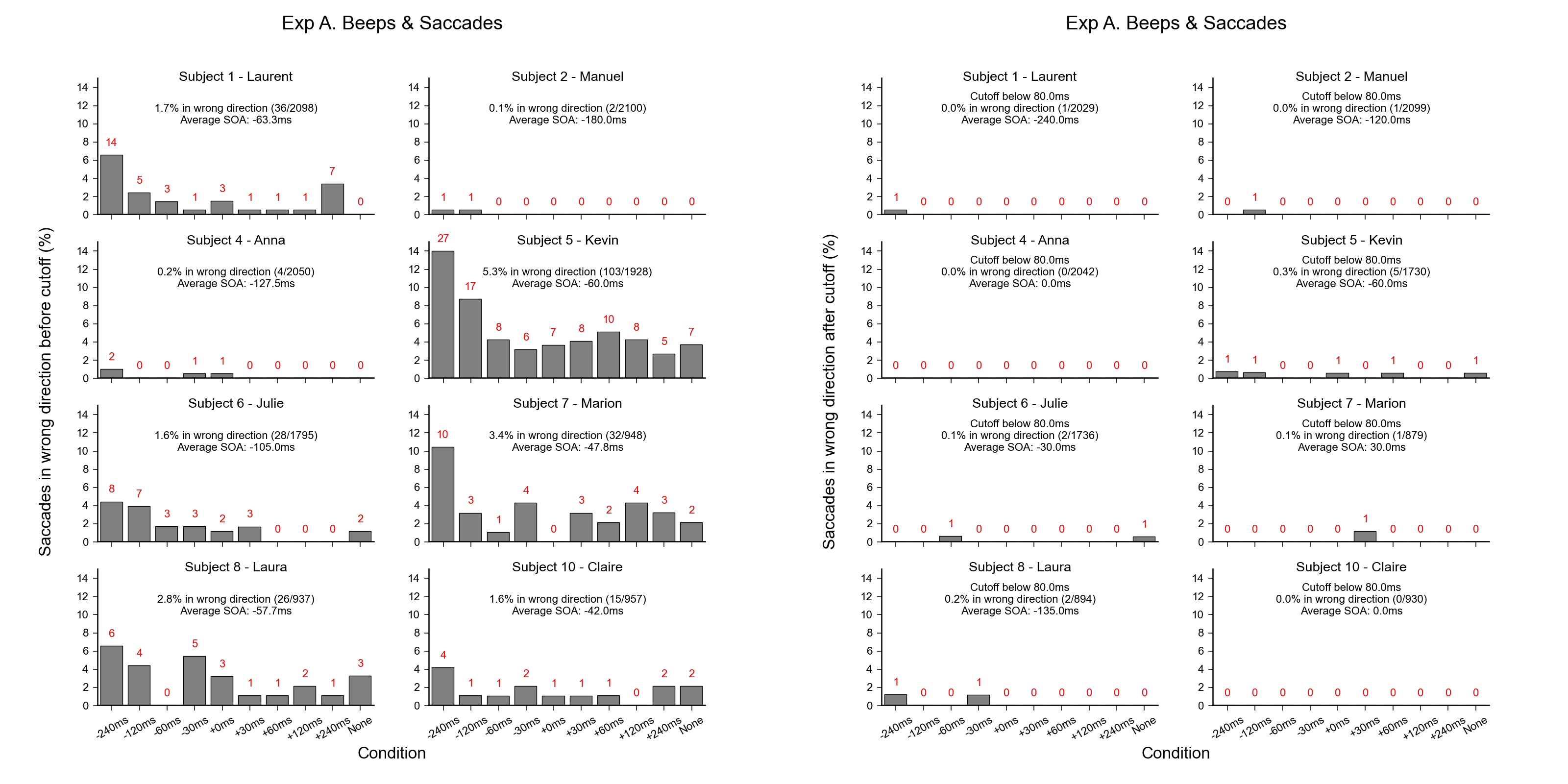

Supplement: S1 Fig — Individual histograms plotting the number of initial saccades going in the opposite direction compared to where the target appeared for each SOA and No beep conditions. The left panel shows the initial data with all the correctly detected saccades and the right panel shows the data after removing those with a gain below 0.4 or with latencies below 80 ms or above 400 ms. (TIF) [file pone.0221192.s004.tif]

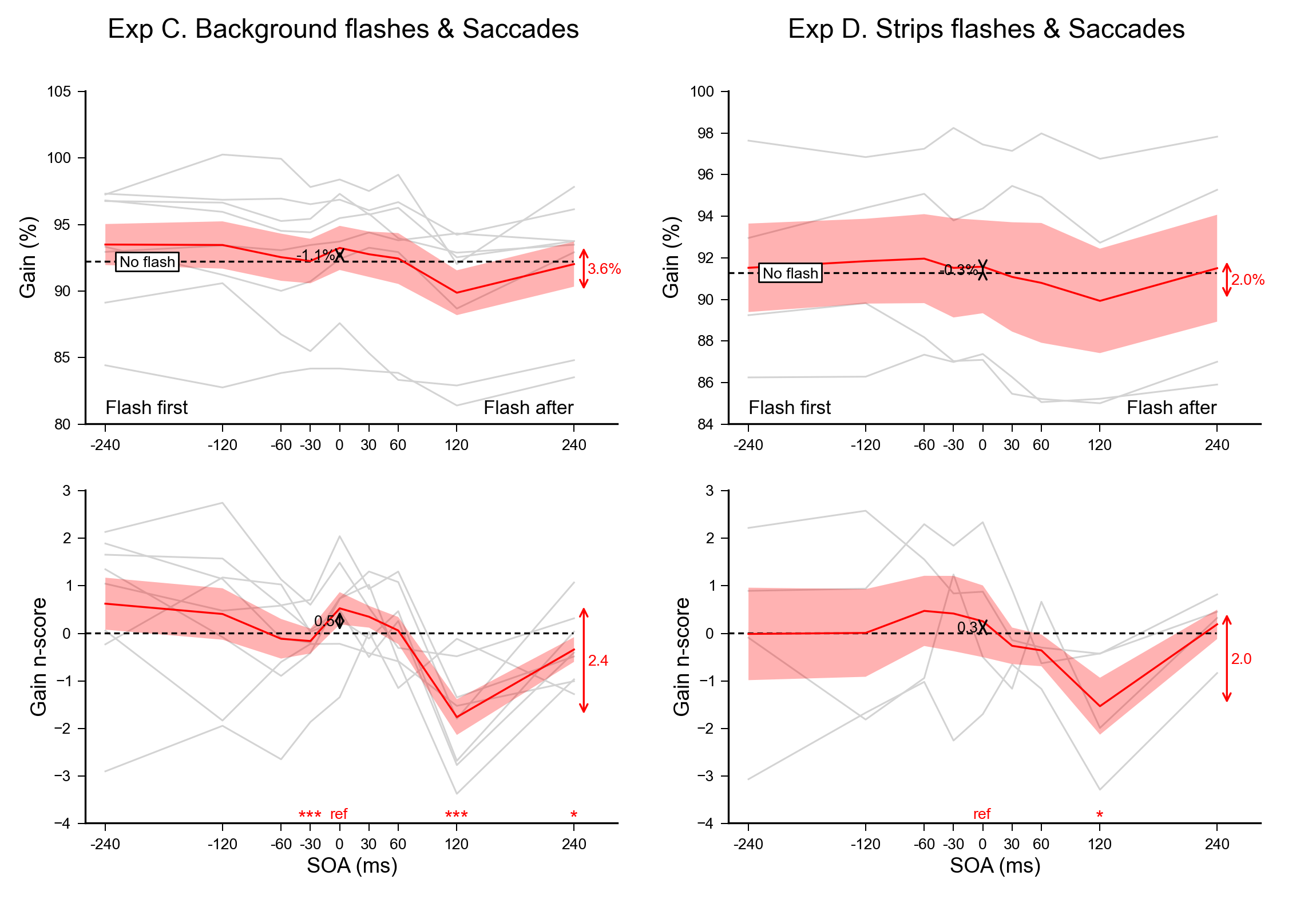

Supplement: S2 Fig — Effect of background flashes (left) and strips flashes (right) on saccadic gain. Saccadic gain averaged across participants for each SOA condition (top) and the corresponding nscores (bottom). Dashed lines show the No flash condition level and grey lines show individual results. Error bars indicate inter-individual SEM. Statistics included a single sample t-test performed on the nscores of the SOA = 0 ms reference condition to highlight the difference with the No flash condition (black arrow), and paired t-tests comparing this reference with each other SOA condition (red stars for each SOA above the X-axis). (TIF) [file pone.0221192.s005.tif]

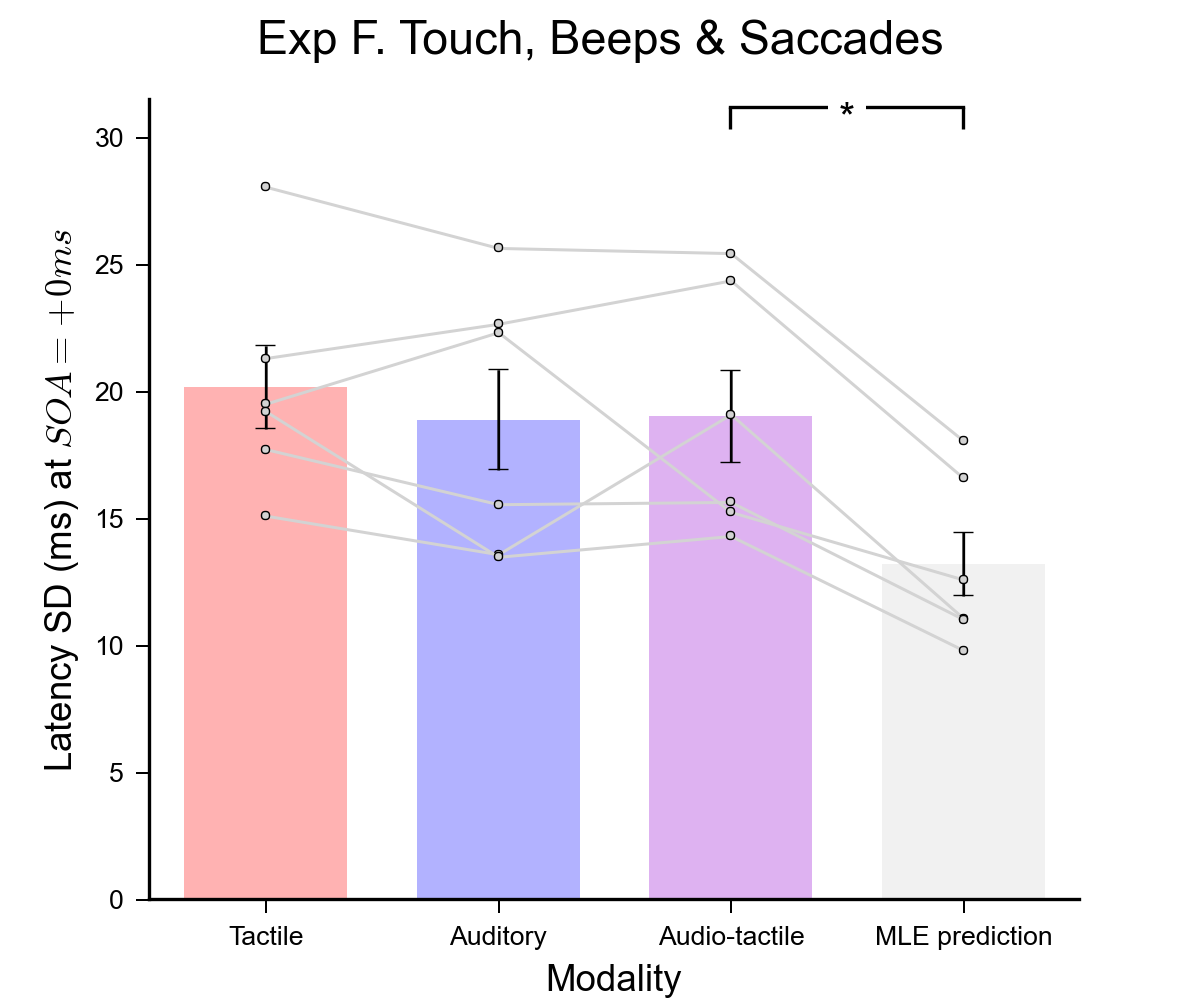

Supplement: S3 Fig — Average latency standard deviation computed when SOA = 0 ms for each modality with the associated MLE optimal prediction. Comparisons were done using paired t-tests. (TIF) [file pone.0221192.s006.tif]
