# Supplementary material for: Irrelevant auditory and tactile signals, but not visual signals, interact with the target onset and modulate saccade latencies
Source: PLoS One. 2020 Feb 11;15(2):e0221192. doi: 10.1371/journal.pone.0221192 (PMC7012454; doi:10.1371/journal.pone.0221192)
Supplement: Attachment — Submitted filename: Review of Madelain Plos One.docx [file pone.0221192.s007.docx]

There is an overall scarcity of research papers addressing the processing of multisensory signals. In this contribution, the authors use saccadic eye movements as a model system to study the facilitatory or modulatory role of auditory and tactile signals on visual signal processing, reporting the following key findings: (1) Simultaneous presentation of auditory / tactile signal and visual target resulted in a “warning effect” and shorter saccade latencies. (2) When auditory / tactile signals were not synchronized with visual target presentation, a “modulation effect” occurred which could result in either shorter or longer latencies; a visual signal resulted in increased latencies. (3) A combination of both modalities increased the modulation effect only to a small extent, interpreted as a saturation of the multisensory interaction with oculomotor control. This paper makes an important and novel contribution to our understanding of how we integrate information from different senses to quickly direct the eyes to objects of interest. The paper is well written and overall technically sound, but I have some concerns about the size of the obtained effects based on the small sample size.

Main results for exp. 1 rely on data that are relatively diverse across the small tested subject population (see Fig. 2 upper panels), yet a highly significant latency difference of 11 ms is reported. The authors also describe their findings as homogenous across observers, yet, Fig. 2 paints a different picture. It appears that for a simultaneous presentation there was no latency difference for at least three observers. These differences have to be acknowledged and discussed, and I’d like to see a more detailed analysis regarding different subgroups of observers, in case there are systematic differences. This comment also applies, perhaps to a lesser degree, to the other experiments.

p.2, para.1: the second half of this paragraph discusses temporal perception and even though this is interesting, it is somewhat unrelated to the rationale of the study. In fact, I found it misleading because it sets up the study as something that it does not deliver. There is no measure of perception here, the variable of interest is saccadic reaction time or latency. Whereas it could be argued that saccades are related to perception, there is also evidence that this is not always the case. I would much prefer to see this introduction lead more towards using eye movements as a model system of studying multisensory processing than as a measure of temporal perception (as nicely done on, p.3, para.2, and even more in para.3 on that page and on p.4). My suggestion would be to either rephrase or simply delete the paragraph in question on p.2.

p.7, Data processing: I am surprised that the standard Eyelink saccade detection algorithm was used. From my lab’s experience, this algorithm is not sensitive enough to detect small saccades. Given that saccades are the main dependent variable in this study, and that the authors are looking into sensitively detecting very early saccades as well, I’d like to see either a discussion and justification for using this algorithm, or a comparison of results obtained with a more sensitive algorithm. It is also somewhat unusual to apply a filter to saccade latencies and remove anticipatory / early onsets. I’d like to see a latency distribution. Whereas typical saccade latencies might be long (around 200 ms or more), there are also reports in the literature of short-latency or express saccades with latencies well below 100 ms, and the present paradigm might well have triggered those.

Overall results presentation: since the procedure and results across experiments are similar and reported in parallel fashion, it would be helpful to streamline the results section overall and report results for control or follow-up experiments in more abbreviated form.

Minor

p.3, l.13: correct in-text citations (Sparks without initials), see also l.22 and other places throughout the manuscript

p.5, l.6-7: rephrase sentence, sounds awkward (“because they provide… allowing the quantification of…”)

p.5, Participants: did participants have normal or corrected-to-normal visual acuity, and if yes, was this tested? Why is it important that subjects were right-handed? Did the results of the two authors differ in any way from those obtained from naïve participants? Were participants remunerated for participation?

p.7, l.1&3: replace “totalizing” by “totaling”

Figure 2 and all similar figures: axes labels are almost impossible to read. Please improve the general aesthetics and readability of this figure with larger font sizes and clearer labels.

p.18: stripes and not strips?

p.21, l.11: please provide additional detail on this device and its manufacturer

p.28, para.1, first sentence: I do not think that the presented results can address the question how this information is processed in the brain; results are purely behavioral and should not be overinterpreted

p.28, l.29: reported as 11 ms above

p.30, title: consider rephrasing “openings” to “limitations” or “open questions”

p.30, last sentence: this is a matter of taste, but this sentence does not add much in terms of content as it is speculative
